# Milk Immune Cell Composition in Dromedary Camels With Subclinical Mastitis

**DOI:** 10.3389/fvets.2022.885523

**Published:** 2022-04-14

**Authors:** Gader Abdulaziz Alhafiz, Fatema Hassan Alghatam, Hams Almohammed, Jamal Hussen

**Affiliations:** Department of Microbiology, College of Veterinary Medicine, King Faisal University, Al-Ahsa, Saudi Arabia

**Keywords:** dromedary camel (Camelus dromedarius), subclinical mastitis (SCM), immune cells, flow cytometry, phagocytosis, bacteria

## Abstract

Mastitis represents one of the most important infectious diseases in camels with heavy economic losses due to reduced milk quantity and quality. Balanced immune cell composition and function in the mammary gland are essential for effective immune response to mastitis pathogens. The objective of the present study was to characterize the cellular immune response to subclinical mastitis in the mammary gland of dromedary camels. Therefore, immunostaining and flow cytometry were used to compare the cellular composition, leukocyte phenotype, and cell viability in camel milk from healthy she-camels (*n* = 8) and she-camels with subclinical mastitis (SCM; *n* = 6). In addition, the *ex vivo* phagocytic activity of milk phagocytes was compared between healthy and affected animals. The health status of the mammary gland was evaluated based on the California Mastitis Test (CMT) score. SCM (CMT score of ≥3 in the absence of clinical signs of mastitis) was found in six of the 56 sampled quarters (10.7 %) with only one affected quarter per animal. In comparison to milk from healthy camels, milk from SCM animals showed higher somatic cell count (SCC), higher numbers of CD45+ leukocytes with an expanded fraction of CD172a+ myeloid cells. Within the myeloid cell population, there was an increase in the percentage of granulocytes (CD172a^+^CD14^low^) with a decreased percentage of macrophages (CD172a^+^CD14^high^) in milk from affected animals compared to healthy animals. The decrease in lymphoid cells in SCM milk was mainly due to the decreased fraction of CD4+ helper T cells. Camel SCM was also associated with a stimulated phenotype, increased cell viability, and enhanced phagocytic activity of the milk phagocytes, macrophages and granulocytes. Collectively, the present study identified significant changes in SCC, leukocyte count, phenotype, viability, and function in association with subclinical mastitis in camels. The results of the present study support a better understanding of host-pathogen interaction mechanisms in the camel mammary gland.

## Introduction

Dromedary camels are well-adapted animals to the harsh environment of semiarid and arid zones with the ability to produce milk of valuable quantity (1). The increased reports on the nutritional and health-promoting properties of camel milk resulted in a currently raised interest in camel milk with growing market demand (2, 3).

Like other dairy animals, camels may be affected by all types of mastitis (4–7). Mastitis represents an inflammatory disease of the mammary gland mainly caused by bacterial pathogens. Unlike the clinical form of the disease, subclinical mastitis is difficult to be detected, because it does not cause any visible changes in milk or udder appearance, like swelling and redness (8). In addition to its effects on animal health and welfare, subclinical mastitis is associated with huge economic losses due to the reduced milk yield and quality and high treatment costs. Moreover, it is a public health concern for camel milk consumers (9).

The characterization of the innate and adaptive immune response of the mammary gland to invading pathogens is essential for the prevention and control of mastitis (10). In addition to milk immunoglobulins and other humoral immune factors, the mammary gland is equipped with several innate and adaptive immune cells that orchestrate the immune response to mastitis pathogens (11–14). The cellular content of the mammary gland secretions, which is called the somatic cell count (SCC), consists of a complex network of cells including neutrophils, macrophages, lymphocytes, and epithelial cells (15, 16). The contribution of the different cell types to the cellular compartment of milk differs according to species. While macrophages are the predominant cells in healthy human and bovine milk (17, 18), the majority of cells in sow and goat milk have been identified as milk granulocytes (19–21). The assessment of the cellular composition of milk, including the absolute counting of somatic cells and the differential proportions of immune cell subpopulations is widely accepted as a valuable tool for the evaluation of the health status of the mammary gland (22–24). For milk samples from healthy camels, a broad SCC range has been reported in the literature (25) with SCC values above 125 × 10^3^ cells/ml milk being indicative of mastitis in camel (8, 25).

In contrast to the microscopic evaluation of the cellular composition of milk, which depends on the subjective evaluation of low numbers of milk cells (26, 27), flow cytometry has the advantage of measuring high cell numbers within a short time, maximizing repeatability of test results. Flow cytometric protocols for the analysis of milk cell composition and viability have been described for several species including cattle (28), goats (19, 20), sheep (29), pigs (21), and humans (30).

In the dromedary camel, several cell surface markers have been recently identified and involved in phenotypic and functional studies. Cluster of differentiation (CD) 45 is a pan-leukocyte marker glycoprotein with tyrosine phosphatase activity involved in the maturation, activation, and differentiation of several immune cells. The hyaluronan receptor CD44 is a type I transmembrane glycoprotein that is expressed on all leukocytes and plays a role in cell–cell interactions and cell migration (31, 32). The surface molecules CD18 and CD11a represent the alpha (α) and beta (β) chains that dimerize to form the adhesion molecule lymphocyte function antigen-1 (LFA-1) (33–35). The signal-regulatory protein alpha (SIRPa; CD172a) is a myeloid cell marker with a regulatory role in several functional activities of myeloid cells (36, 37). CD14, which is mainly expressed on monocytes and macrophages, plays an essential role in the recognition of LPS during infections with Gram-negative bacteria (38). The major histocompatibility complex (MHC) class II, an antigen-presentation receptor, and the scavenger receptor CD163 are commonly used for the analysis of the functional subtype of macrophages (39). CD4 and workshop cluster 1 (WC1) antigen are cell markers that identify helper ab T cells and gd T cells, respectively (35).

Milk phagocytes, including macrophages and neutrophils, are the primary effector cells of the mammary gland innate immune system with a key role during mammary gland infections (17). They contribute to the early elimination of bacterial pathogens by several antimicrobial functions, including phagocytosis, production of reactive oxygen and nitrogen species, and formation of extracellular traps (40).

The mammary gland immune response associated with subclinical mastitis pathogens in camels is still somewhat under-researched in comparison with other dairy animals. The present study was therefore aimed at the comparison of the composition, phenotype, viability, and antimicrobial functions of milk leukocytes from healthy camels and camels with subclinical mastitis.

## Materials and Methods

### Animals

Investigations were conducted on milk samples collected from 14 clinically healthy dromedary she-camels during their first 2 months of lactation. The animals were reared on a private camel farm in Al-Ahsa region in eastern Saudi Arabia. All camels were from Al-majaheem camel breed and in their third and fourth lactations. All animal procedures were approved by the Ethics Committee of King Faisal University (Approval No. KFU-REC-2021- DEC -EA000326).

### Clinical Examination and Milk Sampling

Milk samples were collected from all mammary gland quarters during the evening milking. Before sampling, the udder was palpated and checked for signs of clinical mastitis, such as heat, swelling and pain in the infected quarters, and abnormal alteration in milk color and consistency (41). After discarding the first milk jets, teat ends were cleaned and disinfected and about 10 ml milk were collected into sterile glass tubes for the California Mastitis Test (CMT), microbiological analysis, and SCC. Another 100 ml of milk were milked into sterile glass bottles for cell separation and flow cytometry. The health status of the animals was classified as healthy or mastitic based on the results of the CMT (41, 42). For this, 3 ml of each quarter milk were added to an equal amount of CMT fluid and the mixture was rotated by circular movement. The reactions were graded according to the Scandinavian scoring system as previously described (41). A score of 1 was given if there was no visible thickening of the mixture; score 2 represented slight slime which tends to disappear with continued swirling; score 3 indicated distinct slime but without gel formation; score 4 represented the immediate formation of gel that moves as a mass during swirling; score 5 was given if the gel developed a convex surface and adhered to the bottom of the paddle. Animals with milk samples of a test score equal to or more than 3 in the absence of clinical signs of mastitis were classified as subclinical mastitis animals. For healthy she-camels (*n* = 8 animals) with a test score of <3 and no clinical signs of mastitis, pooled composite milk samples representing all four quarters were prepared for flow cytometry. In the affected group (*n* = 6 animals), only milk samples collected from the affected quarters were further processed for SCC and flow cytometry. Collected milk samples were kept in a cool box and were further processed in the lab within 4 h from the time of collection.

### Somatic Cell Count

Milk SCC was performed after fat globule removal by the spin-wash method (43). Milk samples (500 μl) were diluted with 500 μl PBS in a 1.5 ml tube and the diluted samples were centrifuged at 1,000 × g for 2 min. The upper cream layer was removed using a cotton swab and the remaining skim layer was poured off. For the second wash, 1 ml PBS was added to the tube without resuspending the pellet. The washing step was repeated twice. After the final wash, the cell pellet was resuspended in 500 μl PBS by gently pipetting up and down. The washed cell suspension (100 μl) was stained to an equal volume of Turk solution, which stains the cell nuclei blue, and the SCC was performed using Neubauer counter and light microscopy (44).

### Bacteriological Analysis

For bacteriological analysis, 10 μl of milk were streaked on blood agar and MacConkey agar plates, and were incubated for 24-48 h at 37°C. The plates were then examined for growth colony morphology. Individual colonies were picked for microscopic identification using Gram staining (45). Briefly, thin smears were prepared from the plate cultures, allowed to air dry, and then fixed with heat. Smears were covered with crystal violet solution for 1 min followed by gentle rinsing with water. After that, the smears were covered with Gram iodine solution for 1 min followed by rinsing with water. After that, decolorizer solution was added to the smears for 20 s. Finally, counter-staining with safranin solution was performed for 1 min followed by rinsing with water. The smears were examined microscopically at 1,000 × magnification with oil immersion. The bacterial species were identified based on the shape, arrangement and gram reaction of the organisms as previously described (46).

### Cell Separation

Eight milk samples collected from eight healthy animals (each representing four quarter milk samples) and six milk samples collected from affected quarters of six affected animals were used for cell separation and flow cytometry. Separation of milk cells was performed according to a method previously described for caprine milk (47) with some modifications. Briefly, milk samples were diluted with cold PBS (25 ml milk and 25 ml PBS) in conical 50 ml polypropylene tubes and the tubes were centrifuged at 800 × g and 4°C for 20 min without brake. After removing the fat layer using a spatula, the supernatant was discarded. The cell pellet was resuspended with 30 ml cold PBS and washed twice at 600 × g and 4°C for 10 min. For parallel staining of blood leukocytes, leukocytes were separated from one EDTA blood sample collected from a healthy dromedary camel as previously described (48). Concisely, 1 ml blood was incubated with 5 ml distilled water for 20 s followed by the addition of 2 × PBS to restore tonicity. After centrifugation (1,000 × g, 15 min, 10°C, without brake), the supernatant was discarded and the washing step was repeated twice (at 500 × g and 250 × g for 10 min) until complete removal of red blood cells. Blood and milk cell pellets were suspended in 1 ml cell staining buffer (PBS containing 5 g/l BSA, 100 mg/l NaN3) at concentrations of 5 × 10^6^ cells/ml. Cell viability was determined after incubating the cells with the nucleic acid stain propidium iodide (PI; 2 μg/ml, BD Biosciences, Germany).

### Antibodies

The monoclonal and polyclonal antibodies used are shown in Table 1 (44, 48, 49). Leukocytes were identified using a monoclonal mouse antibody against lama pan-leukocyte marker CD45 (clone LT12A). A monoclonal antibody to the myeloid cell marker signal-regulatory protein alpha (SIRPα), also called CD172a (clone DH59b), was used to differentiate myeloid cells from lymphoid cells. Macrophages and granulocytes were differentiated based on their differential expression of the lipopolysaccharide receptor CD14, which was detected using a mouse anti CD14 monoclonal antibody (clone Tuk4). The antibodies used for helper T cells and gamma delta T cells were mouse anti bovine CD4 (clone GC50A1), and WC1 (clone BAQ128A) antibodies. The monoclonal antibodies to major histocompatibility complex (MHC) class II (clone TH81A5) and the scavenger receptor CD163 (clone LND68A) were used for the analysis of macrophages phenotype. Furthermore, the monoclonal antibodies to CD18 (clone 6.7), CD11a (clone HUH73A), and CD44 (LT41A) were used to measure the expression of selected cell adhesion molecules. All antibodies were tested for reactivity against camel leukocytes in previous studies (35, 50–53). Secondary antibodies to mouse primary antibodies were goat anti-mouse IgM conjugated with Allophycocyanin (APC), goat anti-mouse IgG1 conjugated with fluorescein isothiocyanate (FITC), and goat anti-mouse IgG2a conjugated with phycoerythrin (PE).

**Table 1:** List of monoclonal and polyclonal antibodies.

**Antigen**	**Antibody clone**	**Labeling**	**Source**	**Isotype**
CD14	CAM36A	-	Kingfisher	Mouse IgG1
CD14	Tuk4	APC	Thermofisher	Mouse IgG2a
MHCII	TH81A5	-	Kingfisher	Mouse IgG2a
CD172a	DH59b		Kingfisher	Mouse IgG1
CD163	LND68A	-	Kingfisher	Mouse IgG1
CD4	GC50A1	-	VMRD,	Mouse IgM
WC1	BAQ128A	-	VMRD,	Mouse IgG1
CD11a	HUH73A	-	Kingfisher	Mouse IgG1
CD18	6.7	FITC	BD	Mouse IgG2a
CD44	LT41A	-	Kingfisher	Mouse IgG2a
CD45	LT12A	-	Kingfisher	Mouse IgG2a
Mouse IgM	poly	APC	Thermofisher	Goat IgG
Mouse IgG1	poly	FITC	Thermofisher	Goat IgG
Mouse IgG2a	poly	PE	Thermofisher	Goat IgG

*MHC, Major Histocompatibility Complex; WC1, workshopcluster 1; APC, Allophycocyanin; FITC, Fluorescein isothiocyanate; PE, Phycoerythrin; poly, polyclonal*.

### Cell Labeling and Flow Cytometry

Cell labeling was performed in a three steps staining procedure in a round-bottomed 96-well microtiter plate as previously described (47). For this, 100 μl of the leukocyte suspension (5 × 10^5^ leukocytes per well) of each sample were pipetted into the well of a microtiter plate. After short centrifugation of the plate for 3 min at 300 × g and 4°C, the supernatant was discarded and the primary antibodies (Table 1) diluted in staining buffer were added to the wells. After 15 min of incubation at 4°C, the cell suspension was washed with staining buffer for 3 min at 300 × g and 4°C. In the second staining step, fluorochrome-conjugated secondary antibodies (Table 1) to mouse primary antibodies were added to the wells and the plate was incubated for an additional 15 min at 4°C in the dark. Subsequently, the cells were washed twice with staining buffer for 3 min at 300 × g and 4°C. In the final staining step, an anti-CD14 monoclonal antibody conjugated with APC was added to selected wells followed by a 15 min incubation at 4°C in the dark. Finally, the cells were washed twice with staining buffer for 3 min at 300 × g and 4°C and resuspended in 150 μl staining buffer for flow cytometry. Staining with only antibody isotype controls was included. Labeled cells were analyzed on a FACSCalibur (BD Biosciences) by the acquisition of at least 100 000 total cells. Collected flow cytometric data were analyzed using the FCS Express Software (V3; BD Biosciences).

### Phagocytosis Activity of Milk Phagocytes

Bacterial phagocytosis by milk phagocytes was performed using heat-killed *staphylococcus aureus* (*S. aureus*) bacteria (Pansorbin, Calbiochem, Merck, Nottingham, UK) labeled with fluoresceinisothiocyanate (FITC, Sigma-Aldrich, St. Louis, Missouri, USA) (54). Separated milk cells (2 × 10^5^ in 100 μl RPMI medium) were incubated with *S. aureus-FITC* (50 bacteria/cell) for 45 min at 37°C in 96 well plates. After incubation, the plate was washed in RPMI medium (300 × g for 3 min) and the cells were resuspended in 150 μl of staining buffer and analyzed by flow cytometry. After the identification of the phagocyte population including granulocytes and macrophages, the percentage of phagocytosis-positive cells, as well as their mean green fluorescence intensity (MFI), were calculated.

### Statistical Analyses

Data were processed with the Microsoft office Excel^®^ program (version 2016 Microsoft) and statistical analysis was performed using the software program Prism (GraphPad software version 5, GraphPad Software, San Diego, USA). The Kolmogorov-Smirnov test (with the Dallal-Wilkinson-Lilliefor *P*-value) was performed to check the normal distribution of data. For normal-distributed data, the unpaired student's *t*-test was used to compare the mean of the two groups. For the data that failed to pass the normality test, the non-parametric Mann-Whitney test was used to compare the means. The comparison between milk granulocytes, macrophages, and lymphocytes regarding their phenotype was performed using a one-factorial analysis of variance (ANOVA) with Bonferroni's Multiple Comparison Test. The results for each analyzed parameter were presented graphically as means ± standard error of the mean (SEM). Results were considered statistically significant if the *p*-value was <0.05.

## Results

### Flow Cytometric Identification of Camel Milk Leukocyte Subpopulations

**Figure 1:**
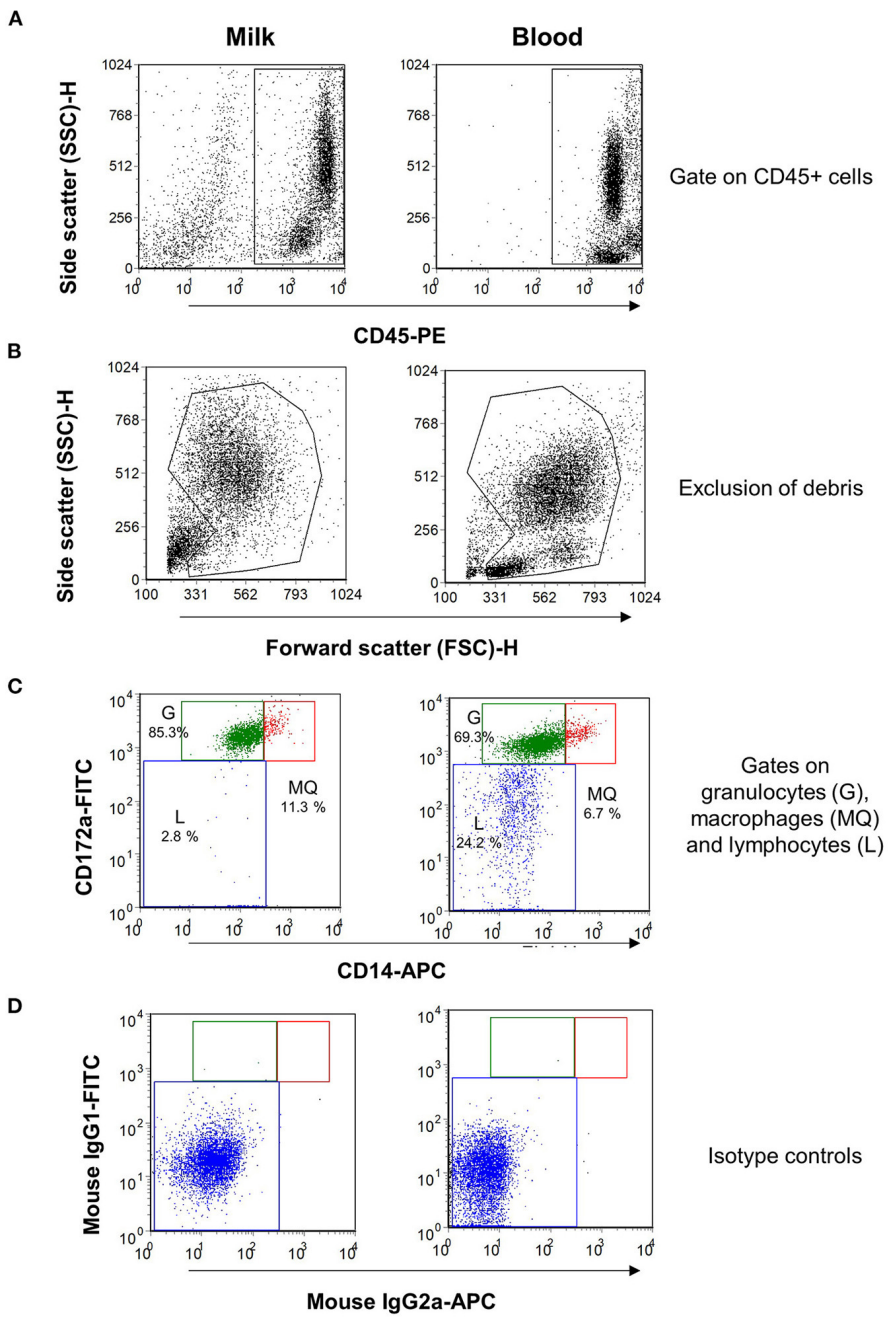
Flow cytometric analysis of leukocytes in milk and blood from dromedary camel. Separated milk and blood leukocytes were labeled with monoclonal antibodies to CD45, CD172a, and CD14, and stained cells were analyzed by flow cytometry. **(A)** Using an SSC/CD45 gate, milk, and blood leukocytes were identified as CD45-positive cells. **(B)** Cell debris were excluded in an FSC-H/SSC-H dot plot. **(C)** Milk and blood granulocytes (G), macrophages (MQ), and lymphocytes (L) were identified based on their staining with monoclonal antibodies to CD172a, and CD14. **(D)** Cells stained with mouse IgG1 and IgM isotype controls.

Using monoclonal antibodies to the leukocyte antigens CD172a and CD14, camel granulocytes, macrophages, and lymphocytes were identified in camel milk samples by flow cytometry. To confirm the expression pattern of the cell surface molecules, blood leukocytes were separated from one animal and were stained with the same combination of monoclonal antibodies (Figure 1). Camel milk granulocytes were identified based on their positive staining with the myeloid marker CD172a and low staining with CD14 (CD172a^+^CD14^low^), while milk macrophages expressed both markers in high levels (CD172a^+^CD14^high^). On the other hand, milk lymphocytes were identified based on their negative staining with the myeloid marker CD172a and the monocytic marker CD14 (CD172a^−^CD14^−^).

### The Immunophenotype of Camel Milk Leukocyte Subpopulations

**Figure 2:**
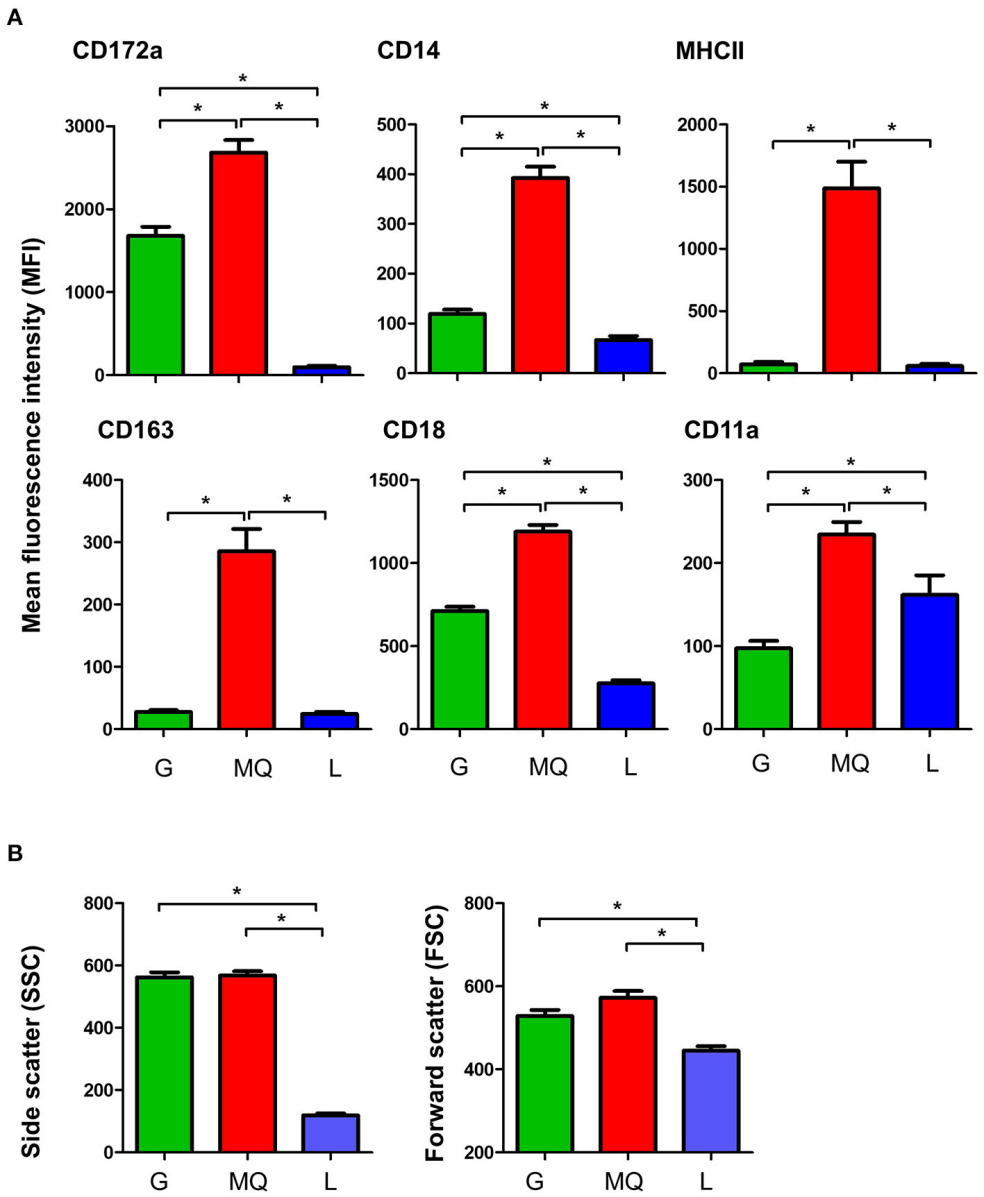
**(A)** The expression levels of the cell surface antigens, CD172a, CD14, MHCII, CD163, CD18, and CD11a on milk granulocyte (G), macrophages (MQ), and lymphocytes (L) as measured by flow cytometry. **(B)** Forward and side scatter characteristics of milk granulocytes (G), macrophages (MQ), and lymphocytes (L) as measured by flow cytometry. *indicates statistically-significant difference between the means.

Milk macrophages showed the highest abundance (*p* < 0.05) of the myeloid marker CD172a (SIRP-alpha), the lipopolysaccharide receptor CD14, the scavenger receptor CD163, the antigen presentation receptor major histocompatibility complex (MHC) II molecules, the cell adhesion molecules CD18 (integrin beta chain), and CD11a (the alpha chain of the lymphocyte function-associated antigen 1; LFA-1) when compared to granulocytes and lymphocytes (Figure 2A). While granulocytes displayed an intermediate level of CD172a, CD14, CD163, MHCII, CD18, and CD11a, lymphocytes showed the lowest abundance of all those molecules, in comparison to granulocytes and macrophages (Figure 2A).

The analysis of light scatter characteristics of milk leukocyte subpopulations revealed similar (*p* > 0.05) cell size (as measured by the forward scatter; FSC) and granularity (as measured by side scatter; SSC) for milk granulocytes and macrophages (FSC^high^SSC^high^), while milk lymphocytes were identified as smaller cells with lower granularity (FSC^low^SSC^low^), in comparison to granulocytes and macrophages (*p* < 0.05) (Figure 2B).

### Subclinical Mastitis

Subclinical mastitis (SCM) was diagnosed based on the California Mastitis Test (CMT) score and the absence of signs of clinical mastitis. SCM (CMT score of ≥3 in the absence of clinical signs of mastitis) was found in six of the 56 sampled quarters (10.7%). All affected camels had only 1 quarter with SCM. The bacteriological analysis identified bacterial cultures in all milk samples collected from the six SCM quarters and only in one sample collected from healthy animals. While two milk samples yielded single bacterial cultures with *staphylococcus* or *streptococcus* species, the other five samples yielded mixed bacterial cultures with *streptococcus* and *staphylococcus* (three samples) or *streptococcus* and coliform bacteria (two samples).

### Somatic Cell Count, Total and Differential Leukocyte Composition in Milk Samples From Healthy Camels and Camels With Subclinical Mastitis

**Figure 3:**
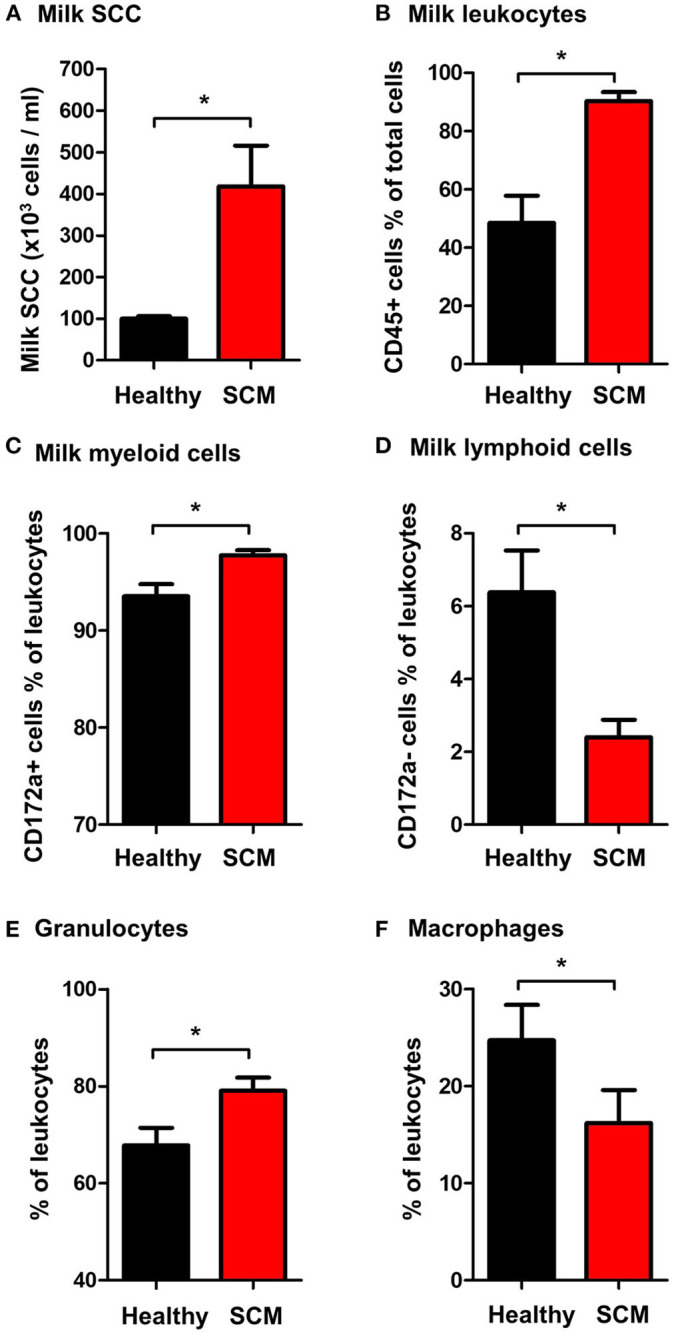
**(A)** Somatic cell count (SCC) in milk samples from healthy camels and camels with subclinical mastitis (SCM). Milk SCC was counted using the direct microscopic method after fat globule removal by a spin-wash followed by staining with Turk solution and microscopic counting on a Neubauer cell counter. The percentage of **(B)** leukocytes (CD45+ cells % of total milk cells), **(C)** myeloid cells (CD172a+ cells % of total leukocytes), **(D)** lymphoid cells (CD172a- cells % of total leukocytes), **(E)** granulocytes (CD172a+ CD14low cells % of total leukocytes), and **(F)** macrophages (CD172a+ CD14high cells % of total leukocytes) in milk samples from healthy and SCM camels as identified by flow cytometry after labeling milk cells with antibodies to CD45, CD172a, and CD14. *indicates significant differences between the means with *p*-values < 0.05.

The SCC, the fraction of milk leukocytes (CD45+ cells), and the differential leukocyte composition were compared between the healthy and SCM animals. The SCC was significantly (*p* = 0.001) higher in milk samples from SCM camels (418.3 × 10^3^ cell/ ml) than healthy animals (103.8 × 10^3^ cell/ml) (Figure 3A). Milk samples with SCM contained higher percentages (*p* = 0.001) of total leukocytes (90.3 ± 3.1% of total cells) than healthy (48.5 ± 9.3% of total cells) milk samples (Figure 3B). The fraction of myeloid cells within the leukocyte population was also significantly elevated (*p* = 0.01) in SCM milk samples (97.8 ± 0.5% of leukocytes) compared to healthy milk samples (93.5 ± 1.3% of leukocytes) (Figure 3C), while the fraction of lymphoid cells was significantly (*p* = 0.01) lower in SCM than in healthy milk samples (Figure 3D). Similarly, the fraction of milk granulocytes was significantly (*p* = 0.02) expanded in SCM samples (79.1 ± 2.7% of leukocytes vs. 67.8 ± 3.7% of leukocytes in healthy milk) (Figure 3E), while the fraction of macrophages was decreased (16.2 ± 3.4% of leukocytes vs. 24.7 ± 3.6% of leukocytes in healthy milk; *p* = 0.04) (Figure 3F).

### Impact of Subclinical Mastitis on Milk Leukocyte Viability

**Figure 4:**
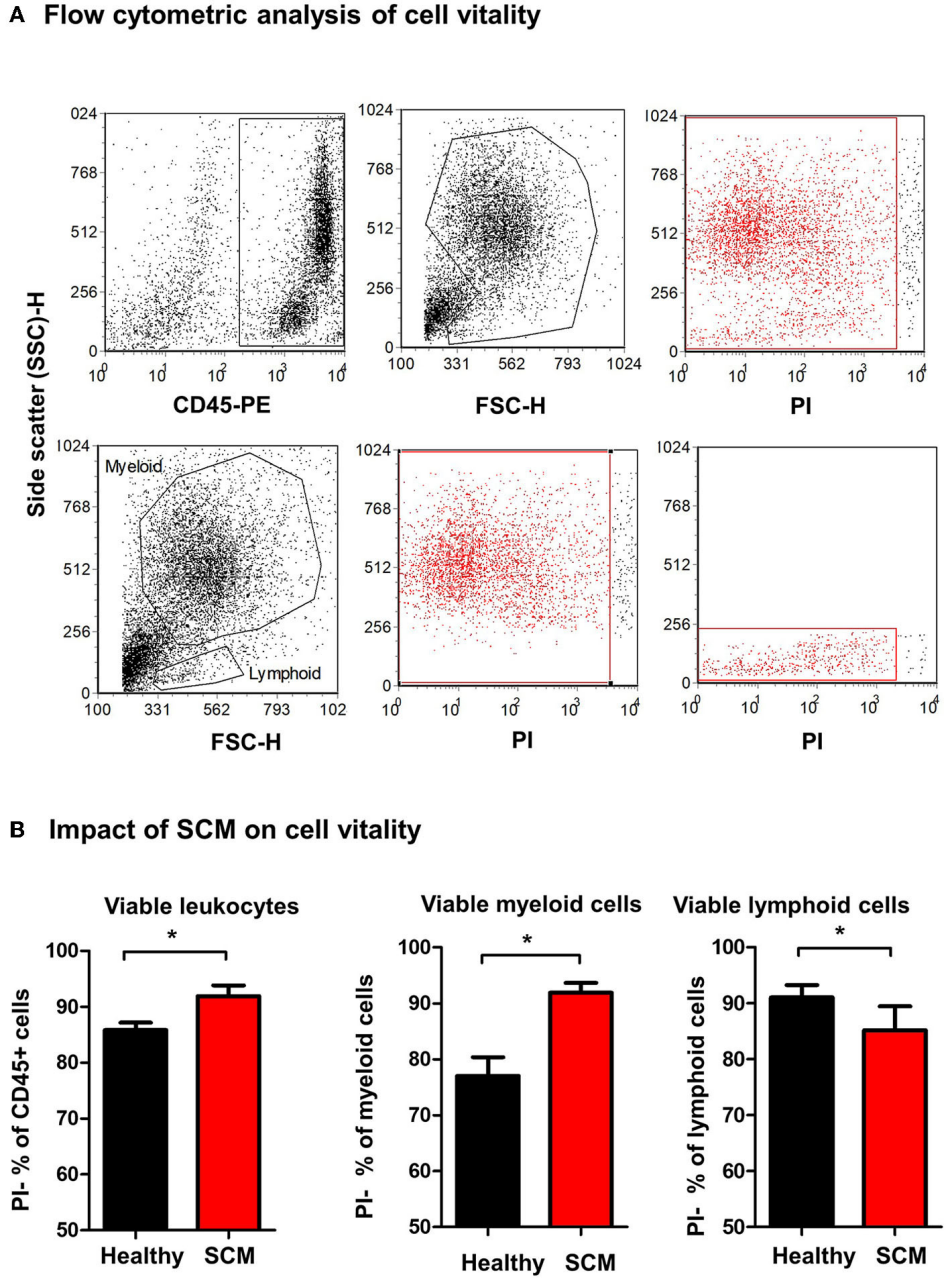
Separated milk cells were labeled with CD45 antibodies and labeled cells were loaded with the nucleic acid stain propidium iodide and analyzed by flow cytometry. **(A)** After gating on leukocytes (CD45 + cells) and the exclusion of cell debris in a SSC-H against FSC-H dot plot, milk phagocytes and lymphocytes were gated based on their FSC and SSC properties. **(B)** The percentage of viable PI-negative cells were calculated in a SSC-H against FL-3 dot plot and the values were presented for total leukocytes, myeloid cells, and lymphoid cells, and presented graphically. *indicates significant differences between the means with *p*-values < 0.05.

The percentage of viable, PI-negative (Figure 4A) milk leukocytes was significantly (*p* = 0.01) higher in milk samples from SCM animals (91.9 ± 1.9% of CD45+ cells) than healthy animals (85.8 ± 1.3% of CD45+ cells). In milk from SCM animals, the myeloid cell population contained a higher (*p* < 0.05) percentage of viable cells (91.9 ± 1.7% vs. 77.0 ± 3.3% of CD172a+ myeloid cells), while the percentage of viable cells under lymphoid cells was significantly lower (*p* < 0.05) in SCM milk (85.2 ± 4.3% vs. 91.0 ± 2.2% of CD172a-lymphoid cells) compared to healthy milk (Figure 4B).

### Milk Lymphocytes From Animals With SCM Contained a Lower Percentage of Helper T Cells

**Figure 5:**
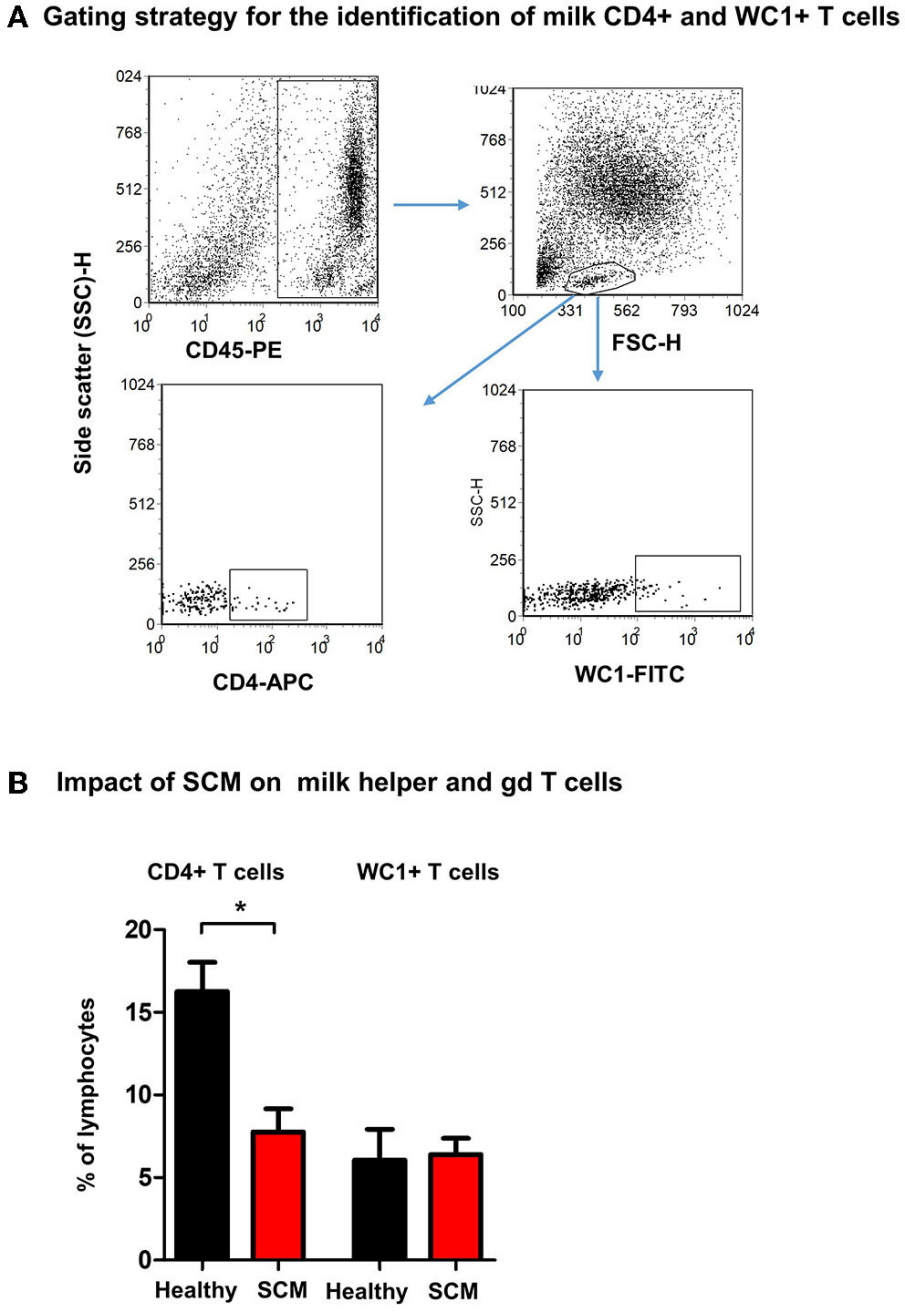
Flow cytometric analysis of milk lymphocyte subsets. Separated milk cells were labeled with monoclonal antibodies to CD45, CD4, and WC1 and analyzed by flow cytometry. **(A)** After setting a gate on milk leukocytes in a CD45 against SSC-H dot plot, lymphocytes were identified based on their SSC and FSC properties. Milk helper T cells and gd T cells were identified within the lymphocyte population based on their staining with anti-CD4 and anti WC1 antibodies, respectively. **(B)** The percentage of T helper cells and gd T cells within the milk lymphocyte population were calculated for healthy and SCM camels and presented graphically. *indicates significant differences between the means with *p*-values < 0.05.

Using monoclonal antibodies to camel CD4 and WC1, the percentages of helper T cells and γδ T cells were analyzed in milk from healthy and SCM animals (Figure 5A). While the fraction of WC1+ γδ T cells was comparable (*p* > 0.05) between milk samples from healthy and SCM animals, the percentage of CD4+ T helper cells was significantly (*p* = 0.002) lower in SCM (7.8 ± 1.4% of lymphocytes) than healthy milk (16.3 ± 1.8% of lymphocytes) (Figure 5B).

### Granulocytes and Macrophages Shape Change in SCM Milk

**Figure 6:**
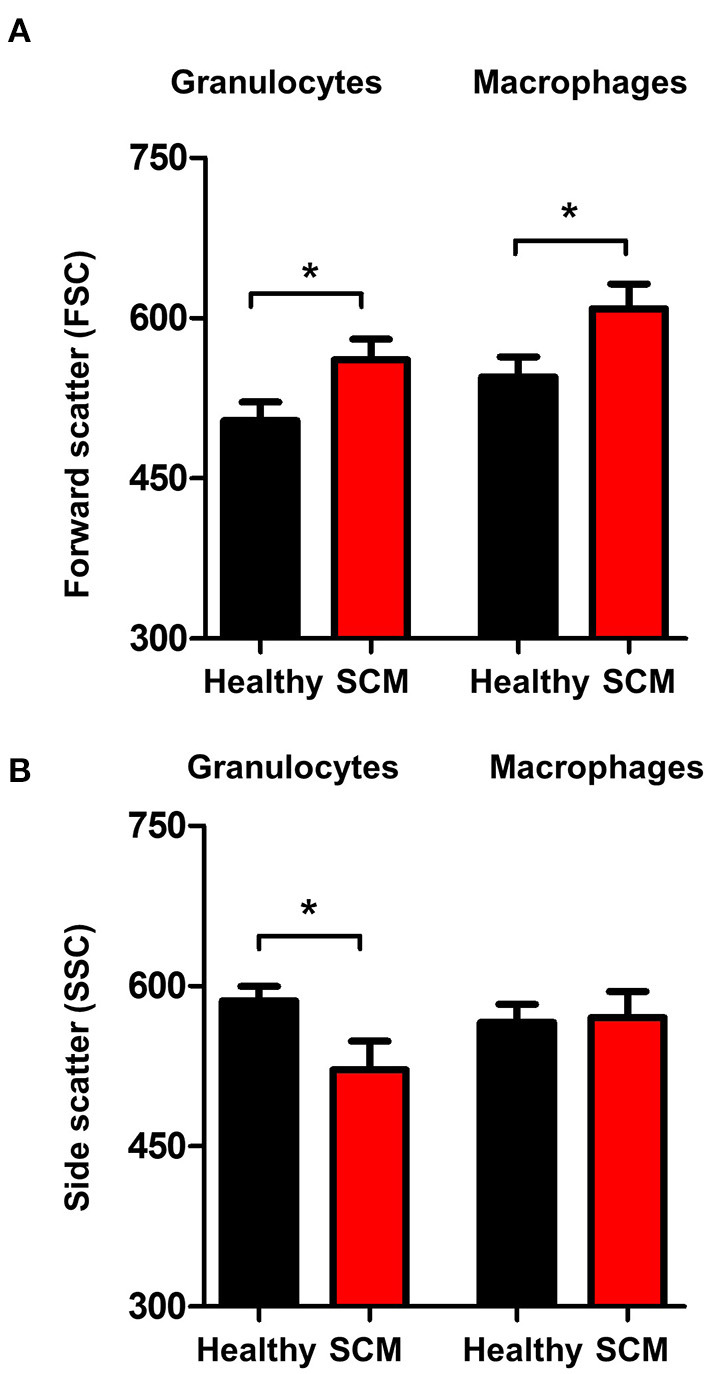
Shape change in milk granulocytes. Milk granulocytes and macrophages were identified based on their staining with CD172a and CD14 antibodies in flow cytometry. Mean FSC **(A)** and SSC **(B)** were calculated and presented for healthy and SCM animals. *indicates significant differences between the means with *p*-values < 0.05.

The analysis of forward scatter (FSC) and side scatter (SSC), which are indicators for cell size and granularity, respectively, revealed higher (*p* < 0.05) FSC values for granulocytes and macrophages from SCM milk compared to healthy milk (Figure 6A). Only for granulocytes, the SSC values were lower (*p* < 0.05) in SCM milk compared to healthy milk (Figure 6B).

### Impact of SCM on the Phenotype of Milk Macrophages

**Figure 7:**
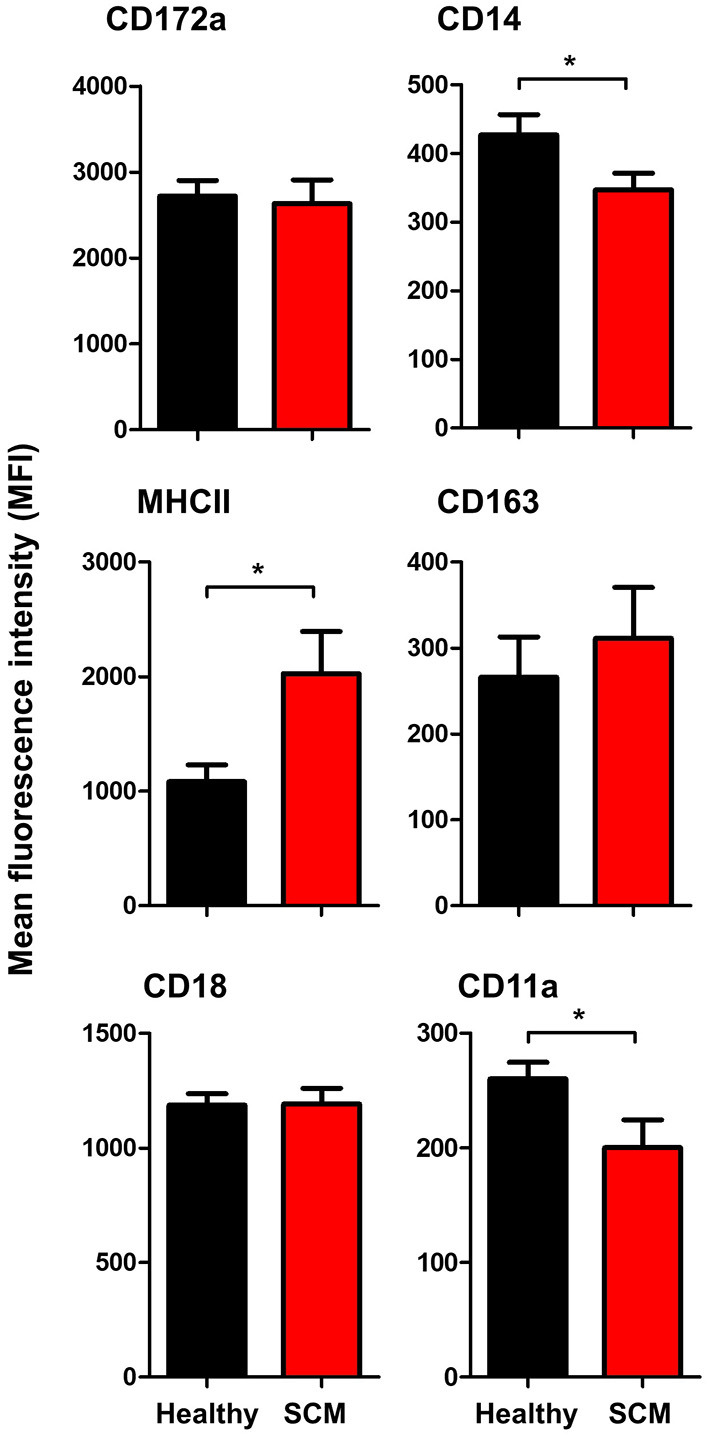
Impact of SCM on the phenotype of milk macrophages. Separated milk cells were stained with monoclonal antibodies to CD172a, CD14, CD163, MHCII, CD18, and CD11a and stained cells were analyzed by flow cytometry. After setting a gate on milk macrophages (based on their higher expression of CD14), the mean fluorescence intensity of the monocytic cell markers and the cell adhesion molecules was calculated and presented for healthy and SCM animals. *indicates significant differences between the means with *p*-values < 0.05.

The comparison between macrophages from SCM and healthy milk regarding their expression levels of several cell surface molecules revealed significant changes in their phenotype. While the abundance of cell surface CD172a, CD163, and CD18 did not differ (*p* < 0.05) between SCM and healthy milk samples, macrophages from SCM milk showed higher levels of MHCII but lower levels of CD14 and CD11a when compared (*p* < 0.05) to macrophages from healthy milk (Figure 7).

### Impact of SCM on the Antimicrobial Function of Milk Phagocytes

**Figure 8:**
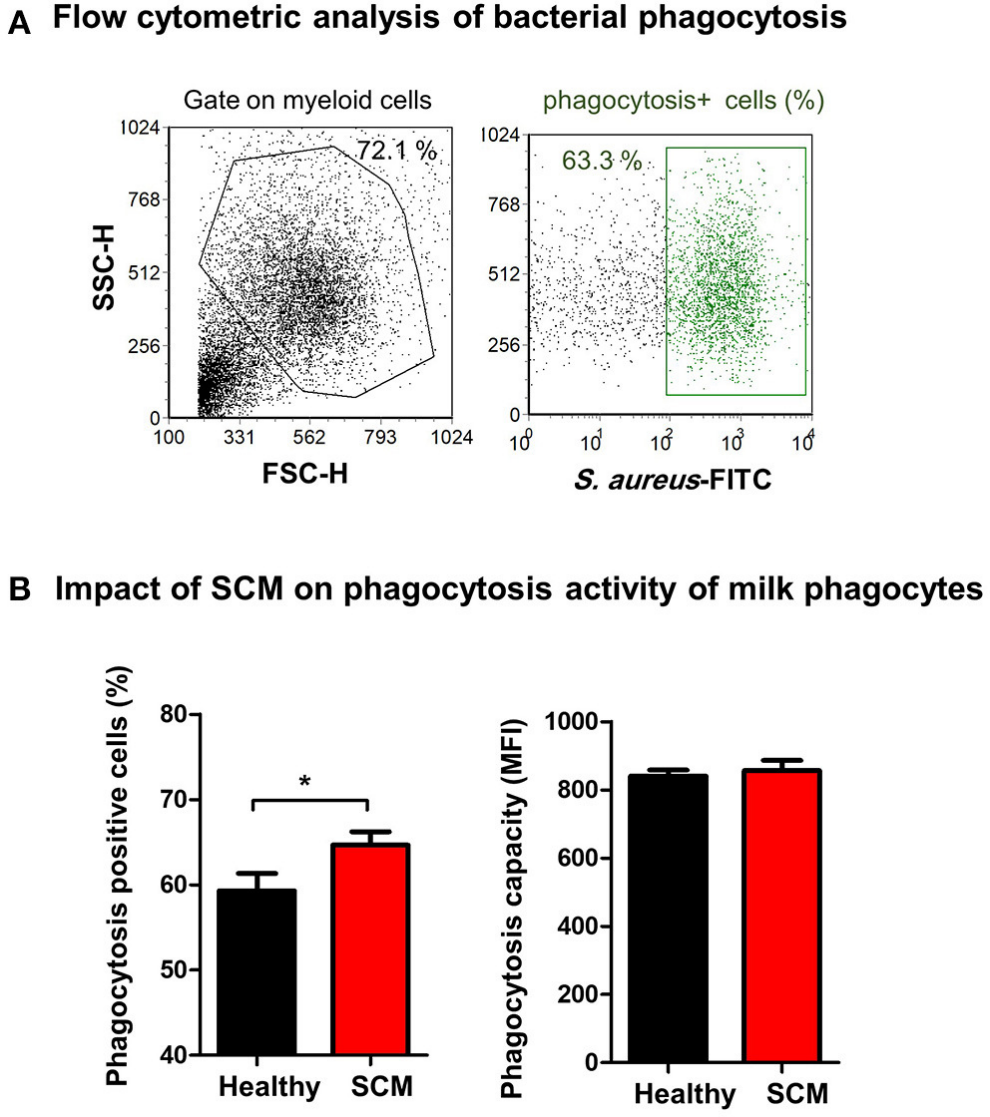
**(A)** Separated milk cells were incubated with FITC-conjugated *S. aureus* and the fraction of phagocytosis-positive cells within the myeloid cell population (including granulocytes and macrophages defined based on SCG/SSC properties) was estimated by flow cytometry based on their enhanced fluorescence in the green fluorescence channel. **(B)** The percentage of phagocytosis-positive cells and their mean fluorescence intensity (MFI), which is a metric of phagocytic capacity of the cells, were calculated and presented for healthy and SCM animals.

The antimicrobial function of milk phagocytes (granulocytes and macrophages) was analyzed by the evaluation of bacterial phagocytosis by flow cytometry (Figure 8A). The percentage of phagocytosis-positive cells was higher (*p* < 0.05) for phagocytes from SCM milk than healthy milk. The phagocytic capacity (the number of bacteria ingested by each cell as measured by MFI of phagocytosis-positive cells), however, did not differ (*p* > 0.05) between cells from SCM and healthy milk (Figure 8B).

## Discussion

The immune cell composition of the mammary gland secretions has been investigated for several animal species in health and disease (19, 20, 55–58). However, studies on the cellular composition of camel milk are limited. The present study employed flow cytometry and monoclonal antibody staining to investigate the differential composition, phenotype, vitality, and some functional aspects of milk leukocytes in clinically healthy camels. The evaluation of the health status of the mammary gland and the classification of the camels was based on the results of the California Mastitis Test (CMT) with a test score of ≥3 in the absence of signs of clinical mastitis being indicative for subclinical mastitis (41).

The present study divided camel milk leukocytes based on their differential expression of the cell surface molecules CD172a and CD14 into a dominant CD172a^+^/CD14^low^/SSC^high^ granulocyte population followed by a smaller fraction of CD172a^+^/CD14l^high^/SSC^high^ macrophages and a minor CD172a^−^/CD14^−^/SSC^low^ lymphocytes population. The higher proportion of granulocytes in camel milk, compared to other species like bovine (17, 28), could be a result of their dominance in peripheral blood (49). The results of the present study, however, largely correspond to data reported for goats regarding the differential composition of milk cells (19, 20). The expression pattern of the cell surface antigens, CD172a, CD14, CD163, MHCII, CD11a, and CD18 on milk granulocytes and macrophages indicates similarities with the immunophenotype of peripheral blood granulocytes and monocytes, respectively, with CD172a, CD18, and CD11a being highest expressed on camel monocytes/macrophages, while MHCII and CD163 being exclusively expressed on monocytes/macrophages (53).

The milk somatic cell count and the CMT are widely accepted tools for the evaluation of the mammary gland health status (17, 28). In camels, elevated SCC values were observed in milk samples from infected mammary glands with values ranging from 1 × 10^5^ to 10 × 10^6^ cells/ml milk (8, 25). In the present study, the four-times elevated SCC with the identification of bacterial cultures in milk samples with a CMT test score ≥3 confirm the results from previous reports regarding the efficiency of CMT and SCC as diagnostic tools for monitoring mammary gland infections in camels.

The two-times increase in whole milk leukocytes with more myeloid cells (CD172a+ cells) in the SCM milk suggests a significant role of the innate immune phagocytes, granulocytes and macrophages, in the immune response to bacterial infections of the camel mammary gland. The increase in the proportion of granulocytes with the decrease in macrophages in SCM milk could be a result of enhanced recruitment of blood neutrophils to the infected mammary gland.

According to reports in dairy cows with bacterial mastitis, the survival of neutrophils was higher in infected than healthy mammary glands (59). The same study identified a link between the higher viability of milk phagocytes with their enhanced antibacterial function. In the present study, the viability of granulocytes and macrophages, as well as the fraction of phagocytosis-positive cells, were higher in SCM milk compared to healthy milk, suggesting similarity in the host-pathogen interaction mechanisms in the mammary gland of cattle and camel.

The lipopolysaccharide (LPS) receptor CD14, the antigen presentation receptor MHCII, and the hemoglobin-haptoglobin receptor CD163 are well-established markers of monocyte and macrophage phenotype (53, 60–62). The differences in the expression levels of MHCII and CD14 on milk macrophages indicate a significant modulatory effect of subclinical mastitis on the functional type of macrophages. Reduced expression of CD14, which plays a key role in the binding of LPS, a cell-wall component of gram-negative bacteria (63–65), may have an impact on the innate recognition function of macrophages in SCM milk.

Macrophages and neutrophils are key effector innate immune cells of the mammary gland with an essential role during the early immune response to mastitis pathogens (17, 40). In the present work, the observed shape-change of granulocytes and macrophages from SCM milk with higher forward scatter values, which correlates with the cell size, and lower side scatter values of granulocytes, which is indicative of cell degranulation (66, 67), indicate the activation status of these phagocytes in the infected mammary gland. This is also supported by the higher fraction of phagocytosis-positive cells in SCM milk.

The lower percentage of helper T cells in SCM milk with no difference in the percentage of γδ T cells suggests a selective impact of bacterial mammary gland infections on T cell subpopulations. As we did not analyze all lymphocyte subsets due to the lack of specific monoclonal antibodies (49), we cannot exclude changes in other milk lymphocyte subsets like CD8+ cytotoxic T cells, B cells, or NK cells in SCM animals.

Collectively, the present study identified significant differences between healthy camels and camels with SCM regarding the cellular composition of their milk. Milk from SCM camels had higher SCC with higher fractions of total leukocytes, myeloid cells, and granulocytes, but reduced fractions of lymphoid cells and macrophages. Within the lymphoid cell population, the percentage of CD4+ T helper cells was reduced in milk from SCM camels. In addition, SCM was associated with improved cell viability and phagocytic activity of milk phagocytes. The results of the present study pave the way for the characterization of the camel immune response to mammary gland infections and support a better understanding of host-pathogen interaction mechanisms on mucosal surfaces in camels.

## Data Availability Statement

The raw data supporting the conclusions of this article will be made available by the authors, without undue reservation.

## Ethics Statement

The animal study was reviewed and approved by Ethics Committee of King Faisal University.

## Author Contributions

GA: sample collection and sample preparation for flow cytometric analysis. FA: sample collection and flow cytometric analysis. HA: flow cytometric analysis and writing the original manuscript. JH: funding acquisition, conceptualization, flow cytometric analysis, and writing the manuscript. All authors have read and approved the final manuscript.

## Funding

This work was supported through the Annual Funding track by the Deanship of Scientific Research, Vice Presidency for Graduate Studies and Scientific Research, King Faisal University, Saudi Arabia (Project No. AN000337).

## Conflict of Interest

The authors declare that the research was conducted in the absence of any commercial or financial relationships that could be construed as a potential conflict of interest.

## Publisher's Note

All claims expressed in this article are solely those of the authors and do not necessarily represent those of their affiliated organizations, or those of the publisher, the editors and the reviewers. Any product that may be evaluated in this article, or claim that may be made by its manufacturer, is not guaranteed or endorsed by the publisher.
